# A comprehensive bibliometric survey of micro-expression recognition system based on deep learning

**DOI:** 10.1016/j.heliyon.2024.e27392

**Published:** 2024-03-04

**Authors:** Adnan Ahmad, Zhao Li, Sheeraz Iqbal, Muhammad Aurangzeb, Irfan Tariq, Ayman Flah, Vojtech Blazek, Lukas Prokop

**Affiliations:** a Key Laboratory of Underwater Acoustic Signal Processing of Ministry of Education, School of Information Science and Engineering, Southeast University, Nanjing, 210096, China; b Department of Electrical Engineering, University of Azad Jammu and Kashmir, Muzaffarabad, 13100, AJK, Pakistan; c School of Electrical Engineering, Southeast University, Nanjing, 210096, China; d College of Engineering, University of Business and Technology (UBT), Jeddah, 21448, Saudi Arabia; e MEU Research Unit, Middle East University, Amman, Jordan; f The Private Higher School of Applied Sciences and Technology of Gabes, University of Gabes, Gabes, Tunisia; g National Engineering School of Gabes, University of Gabes, Gabes, 6029, Tunisia; h ENET Centre, VSB—Technical University of Ostrava, Ostrava, Czech Republic

**Keywords:** Bibliometric analysis, Micro expression, Scopus, Web of science

## Abstract

Micro-expressions (ME) are rapidly occurring expressions that reveal the true emotions that a human being is trying to hide, cover, or suppress. These expressions, which reveal a person's actual feelings, have a broad spectrum of applications in public safety and clinical diagnosis. This study provides a comprehensive review of the area of ME recognition. A bibliometric and network analysis techniques is used to compile all the available literature related to ME recognition. A total of 735 publications from the Web of Science (WOS) and Scopus databases were evaluated from December 2012 to December 2022 using all relevant keywords. The first round of data screening produced some basic information, which was further extracted for citation, coupling, co-authorship, co-occurrence, bibliographic, and co-citation analysis. Additionally, a thematic and descriptive analysis was executed to investigate the content of prior research findings, and research techniques used in the literature. The year wise publications indicated that the published literature between 2012 and 2017 was relatively low but however by 2021, a nearly 24-fold increment made it to 154 publications. The three topmost productive journals and conferences included IEEE Transactions on Affective Computing (n = 20 publications) followed by Neurocomputing (n = 17) and Multimedia tools and applications (n = 15). Zhao G was the most proficient author with 48 publications and the top influential country was China (620 publications). Publications by citations showed that each of the authors acquired citations ranging from 100 to 1225. While publications by organizations indicated that the University of Oulu had the most published papers (n = 51). Deep learning, facial expression recognition, and emotion recognition were among the most frequently used terms. It has been discovered that ME research was primarily classified in the discipline of engineering, with more contribution from China and Malaysia comparatively.

## 1 Introduction

Micro-expressions (ME) are transient, low-intensity facial expressions that occurs when people try to conceal their genuine feelings, either purposefully or subconsciously [1]. As a result, it is difficult to discern such genuine emotional information. One of the most significant concerns is that the duration of a ME is exceedingly short, lasting approximately 0.04s–0.2s [2], and other literatures have found that the duration of a ME is less than 0.33s and does not surpass 0.5s [3]. The rapid arrival and disappearance of ME makes tracking and identification more difficult. Similar to low-intensity ME, they only apply to a portion of facial expression action units [4]. As a result, even without formal instruction, the human eye can quickly recognize ME and most participants struggle to identify it.

Many researchers have been working hard over the past decades to assist computers better interpret human facial ME and emotional engagement. Takalkar et al. [5], documented the literature about face detection and recognition, filtering, facial structure detection and selection, and classification. These methods were categorized into three types: appearance-based methods, motion-based methods, and deep learning-based methods. From which it can be observed that these techniques focused on appearance have garnered greater interest in the literature. These methods describe the dynamics of expression intensity as well as textural details like furrows and wrinkles. To characterize ME in appearance-based methods, a variety of representations have been used, such techniques include Gabor filters, 2D Gabor filter and Sparse Representation (2DGSR), Discriminant Tensor Subspace Analysis (DTSA) and Local Binary Patterns on Three Orthogonal Planes (LBPTOP) [[6], [7], [8], [9], [10], [11], [12]]. Several studies [11,[13], [14], [15], [16]] presented LBPTOP variants to rapidly amplify ME recognition. Guo et al. [13], introduced Three Orthogonal Planes with Centralized Binary Patterns (CBP-TOP). In their work they only considered pixels with the highest weight among their neighbors. Huang et al. [17], proposed Spatio-Temporal Local Binary Patterns with Integral Projection. They employed integral projection to maintain facial image shape attributes and hence improve micro-expression discrimination. Huang et al. [14], went even further, proposing the Spatio-Temporal Completed Local Quantized Patterns (STCLQP). Instead of homogeneous patterns, they used orientation, sign, and magnitude components to construct appropriate pattern codes. The usage of these pattern codes can result in improved performance; however, it is based on the training dataset used to develop classifiers. LBPTOP was used by Zong et al. [15], to detect ME, the data set was preprocessed with Eulerian video magnification (EVM), which involved amplifying ME at low intensity. Liong et al. [16], statically selected different face areas based on the frequency of occurrence of Action Units region-of-interest (ROI-selective). According to Zong et al. [15], the traditional spatial division methods cannot guarantee such acceptable subregions since its grid size is fixed. While using the large subregions may result in noisy data that can interfere with spatiotemporal feature performance, while using small subregions results in a loss of valuable information. They developed a hierarchical division method in which face was divided into subregions of varied dimensions to address this issue. STLBP-IP was used for ME features detection [17].

ME samples was used to improve recognition of the three expressions namely "disgust", "happiness", and "surprise" to address the scarcity of labeled ME instances. A Singular Value Decomposition (SVD) approach was used to implement LBP (reps. LBP-TOP) in order to construct a macro-to-micro transformation model for the detection of face macro-expression (reps. ME) features while Coupled Metric Learning (CML) method was used to represent the common characteristics of ME samples and facial macro-information in their Hot Wheel Patterns (HWP) and HWP-TOP algorithms for modeling face macro and ME [18,19]. The results were then compared to cutting-edge methodologies and it was discovered that using macro-expression data to detect ME, did not result in a substantial boost in efficacy.

In past few years, numerous articles have been published which discussed the motion-based approaches for measuring orientations of the facial component and non-rigid movement [[20], [21], [22], [23], [24]]. To compute the facial motion its adjustments among consecutive frames the usage of the brightness conservation idea, The Histogram of Oriented Optical Flow (HOOF) and its variants were commonly used in motion-based automated algorithms for ME detection. To detect movement changes in ME, Li et al. [24], developed the Histogram of Image Gradient Orientation on Three Orthogonal Planes (HIGO-TOP). Magnification on frames was used in their proposed approaches to make the feature more useful.

From last few years to overcome these problems, deep learning (DL) based method has been propose which achieved high-level feature identification and pattern recognitions simultaneously, the proposed approaches were assessed utilizing a range of deep neural network (DNN) topologies, including the Convolutional Neutral Network (CNN) and the Recurrent Neural Network (RNN). For this instance, Kim et al. [25], employ DL based algorithms to recognize ME. In their proposed approach, CNN was used to identify spatial feature space on select video frames at different expression levels (onset, apex, offset). Further, RNN-derived Long short-term memory (LSTM) network was used to recognize time-dependent features in video sequences. Similarly, Peng et al. [26], came up with a new type of neural network called the "DTSCNN" which stands for Dual Temporal Scale Convolutional Neural Network for ME recognition. In their method of calculating the high-dimensional spatiotemporal space of features, they incorporated optical-flow sequences over various temporal scales into the DTSCNN. In addition, Wang et al. [27], and Reddy et al. [28], proposed an architecture of 3D-CNNs derived from video sequences for the purpose of convolutional and feature identification based on the 3D kernel. Takalkar et al. [29], recently presented a hybrid technique to recognizing ME based on handmade and deep features. The handmade features, which were based on the LBP-TOP, depicted the face's spatio-temporal motions, whilst the deep features were derived using CNN. The process of recognition was performed in a “black box” using Deep Learning (DL) algorithms.

Although the development of DL based algorithms and outstanding classifiers in ME identification [30,31], their dependability in ME detection remains a challenge due to the limited number of ME datasets. To address the issue of insufficient samples in the ME dataset, Zhi et al. [32], and Wang et al. [33], used transfer learning. They use the ME dataset to train their model. The model was then transferred and fine-tuned to recognize ME. The fundamental issue with Transfer learning-based methods is about performance which suffers as a result of noise such as brightness, misaligned face, and the relatively brief duration and subtle movement of ME [34].

The enormous size of the feature space is a significant drawback of appearance-based approaches. For instance, Huang et al. [14], used more than 23,000 features while the method that Wang et al. [11], developed, contained about 4425 features. Wang et al. [34], used Facial Action Coding System (FACS) [35] to establish 16 ROIs in order to solve this issue. Additionally, ROIs were also used for ME detection by Liu et al. [20], and Zong et al., [15]. They demonstrated that ME changes the appearance of only a small number of local and local subregions of the face. Studies have shown that a model's ME efficiency can be increased by including ROI-based features. The pyramid of uniform local binary patterns was then used as the basis for a model developed by Abdallah et al., [36].

It is noteworthy that even after ROIs are used to identify the feature space of appearance-based methods, their effectiveness in differentiating ME is still limited when compared to deep learning-based methods and motion-based methods. We believe that this is due to confusion in the classification of identical ME, which is one of the most difficult challenges for all researchers in this field. In light of the previously discussed literature, there was a sudden offshoot in research and understanding in the ME recognition domain. As a result, the bibliometric analysis is conducted, which aids in a full understanding and grasp of this topic. Bibliometrics is described as a field of study in which scientists evaluate bibliographic data (i.e., published literature) using various statistical and mathematical techniques to discover significant trends and patterns [[37], [38], [39], [40]]. Bibliometric studies assess and measure the influence and impact of publications. These researchers commonly seek out patterns in the production, dissemination, and reception of information, alongside examining the relative significance and impact of diverse bibliographic elements such as citations, authors, institutions, and journals, among other factors [37,38]. A comprehensive and unbiased evaluation of the status of a specific field is a fundamental objective of bibliometrics research. This objective is accomplished by quantifying the volume and caliber of relevant publications, as well as the frequency with which they are acknowledged and utilized by other scholars. Such assessments can be utilized by researchers, policymakers, and funding organizations to identify areas of excellence and areas in need of improvement, enabling them to make well-informed decisions regarding the allocation of resources and support. Additionally, bibliometrics research can be used to identify new trends and potential areas of expansion in a particular field by examining the shift in publishing and citation practices over time. This can help researchers uncover new prospects and subjects while also staying up to date on developments in their respective fields. Bibliometric studies can also be used to examine the reach and influence of specific publications, authors, and institutions. These researches track the number of citations, the journals in which the publications were published, and the nations and institutions that cite the papers. Such evaluations can help researchers evaluate their own work while also uncovering prospective collaborations and networking opportunities with other scholars. Furthermore, corporations can use this type of analysis to make educated decisions and assess the effectiveness of specific organizations, countries, and publications.

Some of the prominent software programs used by researchers to undertake bibliometric analysis include VOSviewer [41], BibExcel [42], CiteSpace [43], and Bibliometrix [44]. Thus, providing data on an open access portal for bibliometric analysis is a solid practice for supporting new researchers. It is imperative for new researchers to remain abreast of developments in their field by providing pertinent analyses, evidence-based descriptions, and precise representations derived from data obtained from the Scopus and Web of Science databases [45].

The purpose of this analysis is to track the evolution of research trends in ME overtime. This includes the identification of emerging topics, shifts in focus, changes in methodologies. Analyzing bibliometric data can reveal the most prominent journals, conference, authors or another ME research is disseminated. This information can be valuable for researchers looking to target specific topic in ME recognitions. This study's findings can be utilized to identify current trend and hot topics of published articles for further study and research. This also provide in-depth understanding of a research topic by inducing and mapping authors' regions. To gain research knowledge, it is necessary to be aware of the existing constraints [46]. This helps discovering new trends as well as exposing the future research, which is the fundamental motivation for this study. To the best of our knowledge, no previous work evaluated the extensive bibliometric literature evaluation and software-based thematic analysis on micro-expression recognition. Furthermore, this study presented a framework for thematic analysis that most researchers and industrial experts can employ in future research. The goal of this study was to provide answers to the following questions.

• What are the research hotspots for ME recognition in the existing literature?
• Which countries, ME-related articles, authors, and/or journals have achieved remarkable results?
• What are the key topics and advancements in the ME recognition research?
• What are the limitations and research priorities in consideration of ME recognition?

In order to carry out an in-depth bibliometrics literature review, R-based tool was used in this study as well as the VOSViewer software. As a result, this study was attempted to improve the following areas of the literature now in existence.

• Our work has established a thematic framework through the utilization of software-based thematic analysis, which has facilitated the investigation of main research hotspot.
• In this study, we additionally classified the preceding research investigations based on the employed methodology. The result provided an insight into current research approaches as well as approaches that have been overlooked.

The article is structured as follows, Section 2 outlines the methodology, data sources, and tools used in the bibliometrics analysis research. Section 3 presents the results and observations, which provides a complete bibliometric study in terms of performance analysis and science mapping. Section 4 presents some limitations of the study. Section 5 presents the conclusions of the research and summarizes its findings.

## 2 Materials and methods

### 2.1 Research methodology

The goal of this study was to perform a bibliometric analysis about ME recognition. The proposed study evaluated published articles by extracting quantitative information from graphical data using a range of statistical techniques (VOSviewer, an R-based tool). Data was gathered using various search engines and analyzed to estimate the number of published articles each year as well as relevant study subjects. It also included information about prominent authors, countries, organizations, and publications in the field of ME recognition. Furthermore, depending on the statistics, the most influential subject areas and articles were highlighted. As a result, our planned bibliometric study has included works on micro facial expression and recognition since the commencement of this survey.

#### 2.1.1 Data collection

In order to obtain bibliometric data, search terms were used in the WOS and Scopus databases on Jan 10, 2023, and the retrieved results were further ranked based on the title, abstract, and other inclusion/exclusion criteria suggested by various investigators.

#### 2.1.2 Identification of search terms

To discover the relevant terms, several publications from the previous literature were reviewed. To locate relevant phrases, Google Scholar was also searched for "micro-expression" and "facial micro-expression." The keywords "facial microexpression" OR "micro-expression" OR "micro expression" OR "facial-microexpression ". A preliminary search was then conducted using the title, abstract and keyword of the publication. This resulted in the detection of 782 articles in Scopus database while the same keywords were also used in WOS and a total of 428 publications were retrieved. The search period was then limited to 2012-22 with only included English-language and peer-reviewed open access works and the final results obtained from Scopus were 712 publications while that of WoS were 404 publications. The search terms were then employed to acquire research and review articles from both WOS and Scopus databases as of January 10th, 2023.

#### 2.1.3 Article selection

The selection of an appropriate database is critical in conducting efficient searches. We made considerable use of the Scopus and WOS data bases in this respect. Scopus, in particular, gives academics greater visibility than other databases, and its sophisticated search functionality tools allow them to organize references and collect citations from papers. Furthermore, the Scopus database includes journals from a wide range of publishers, including Springer, Elsevier, Taylor & Francis, Emerald Insight, and IEEE [43,47]. In addition, WOS was used to fill in the missing articles and journals in Scopus. The first step was to search both databases for relevant phrases. Abstracts, titles, and keywords yielded 712 total results for Scopus and 404 for WOS.

**Fig. 1:**
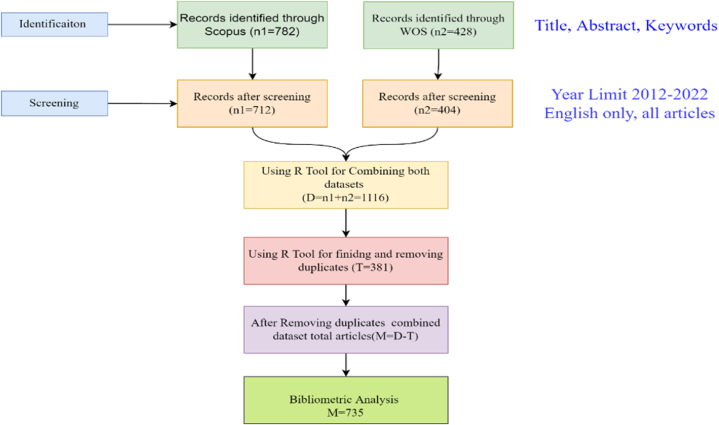
Article selection procedure used in this study.

Fig. 1 depicted the article segment process employed in this investigation. 712 Scopus articles and 404 WOS articles were extracted in two different formats. After combining the two datafiles, R-studio was used to locate and remove identical articles. As a result, 735 articles in Comma Separated Values (CSV) format were exported for bibliometric analysis.

## 3 Results and discussion

### 3.1 Bibliometric analysis

This section addresses the bibliometric analysis of 735 selected publications' author keywords, author partnerships, journals, citations, institutions, theme evaluation, and bibliographic coupling.

#### 3.1.1 Publication by year

**Fig. 2:**
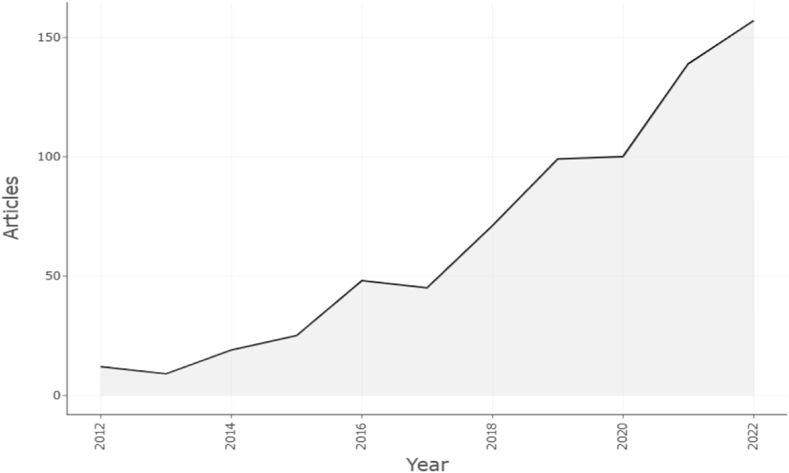
Publication of micro expression recognition per year.

Fig. 2 depicted a significant increase in the publication of research articles over the last ten years, indicating increased interest in micro-expression recognition in academic communities. Fig. 2 showed that the published literature was relatively low between 2012 and 2017. In 2012, there were only 12 published papers; by 2021, a nearly 24-fold increase made it to 154 publications. We anticipated that the publication of micro-expression recognition will grow exponentially in 2023 and subsequent years.

#### 3.1.2 Publication by journals, authors and countries

**Fig. 3:**
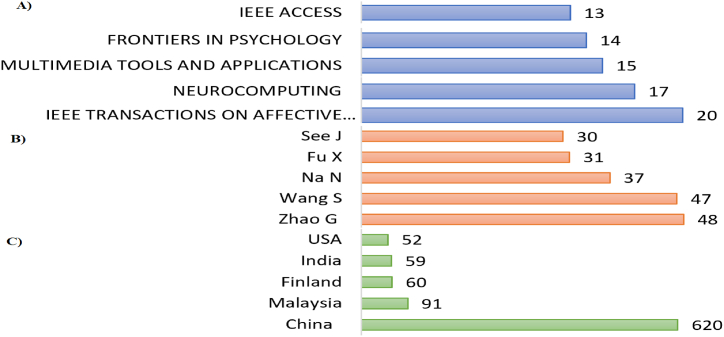
(A) Publication by Journals, (B) Most influential author based on total publications, (C) productive countries.

Fig. 3 showed the top five journals, it is imperative to consider the cumulative quantity of research papers that have been published within the past decade. The three most productive journals and conferences, as shown in Fig. 3a, were IEEE Transactions on Affective Computing (20 papers), Neurocomputing (17 papers), Multimedia tools and applications (15 papers). It was observed that the top three journals together published 41% of the overall literature. Fig. 3b illustrated the top four authors with the most articles published between 2012 and 2022. In addition, the figure revealed that Zhao G was the most proficient author (48 publications) followed by Wang S and NA N with 47 and 37 publications. Likewise, the top influential country as shown in Fig. 3c was China (620 publications) followed by Malaysia (91), and Finland (60) in terms of scientific production.

#### 3.1.3 Publications by citations

**Fig. 4:**
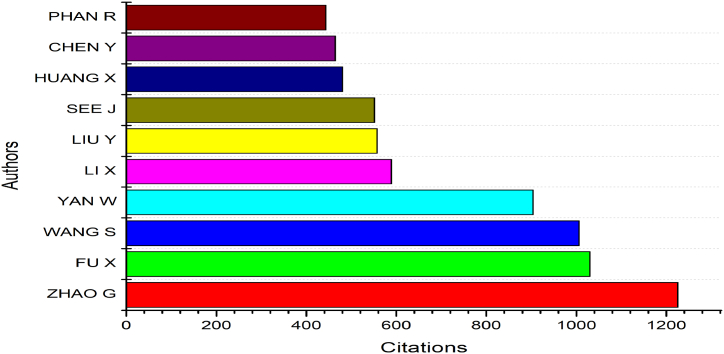
Publication by citations.

The present study evaluated the aggregate number of citations while gathering data and insights on noteworthy authors in the domain of micro-expression recognition. The top ten most frequently cited articles from the Scopus and WOS databases were summarized in Fig. 4. Fig. 4 illustrated that each of the authors acquired citations ranging from 100 to 1225. It is important to note that due to variations in indexing algorithms and time periods employed by different databases, the total number of citations obtained from Google Scholar and other databases may differ. According to Fig. 4, the author Zhao Guoyang's works obtained the most citations (1225). She is currently a full Professor at the University of Oulu's Center for Machine Vision and Signal Analysis (IAPR Fellow, IEEE Senior Member). The documents of author Fu Xiaolan come next, with a total of 1030 citations. She is a Professor of Psychology and the Director of the Chinese Academy of Sciences' Institute of Psychology. She is a pioneer in China's micro-expression study. She has created three open-access micro-expression databases: CASME, CASME II, and CAS (MEU2). Her research interests include lie detection, emotional computing, learning, perception and attention, and perception and attention. Another author with a high number of citations is Wang S. His work has been cited 1006 times, and he is now an Assistant Researcher at the Institute of Psychology of the Chinese Academy of Sciences. He is the author of approximately 40 scholarly papers. At the 2011 International Joint Conference on Biometrics, he is among the ten doctoral consortium participants. Therefore, it is acceptable to believe that all the publications depicted in Fig. 4 were among the most well-known studies regarding recognition of micro-expressions in literature.

#### 3.1.4 Publication by institutions

**Fig. 5:**
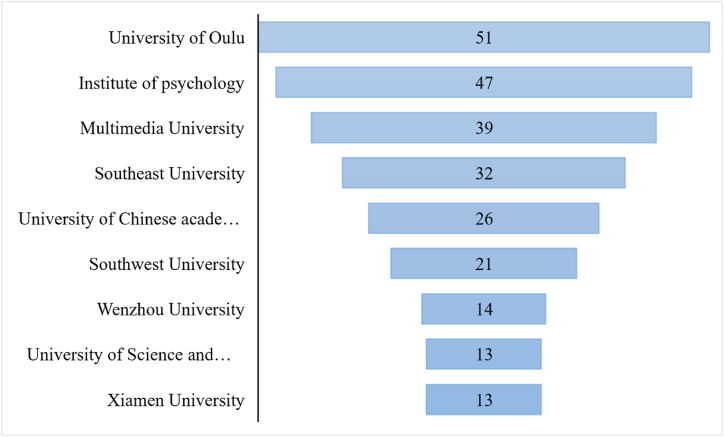
Top nine institutions numbers of publication.

Fig. 5 depicted that the University of Oulu has the most published papers (51), among the top nine the top nine academic institutions. The Institute of Psychology (the Chinese Academy of Sciences) was revealed to be the second ranked with total of 47 publications while the Multimedia University (Malaysia) was ranked third with 39 number of publications. According to the analyzed results, seven of the top ten most productive institutions were from China and actively pursued research into micro-expression recognition.

#### 3.1.5 Common words in title and abstract

**Fig. 6:**
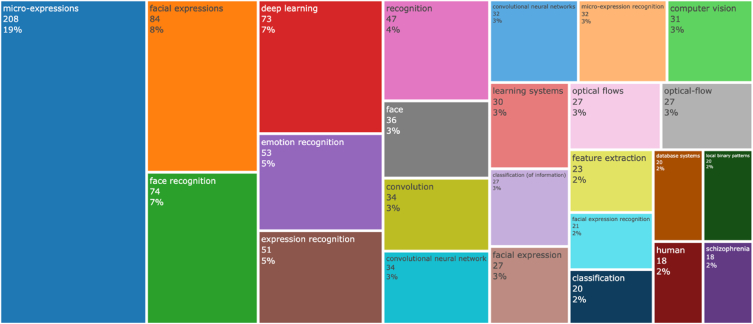
Tree-map of the most commonly used words in ME recognition publications' titles and abstracts.

Fig. 6 illustrated the Common words used in abstracts and titles of all the publications. The most common term used was micro-expressions with 208 times in title-abstracts, followed by facial expressions (84 times), face recognition (74 times), and deep learning (73 times).

#### 3.1.6 Common words in keywords

**Fig. 7:**
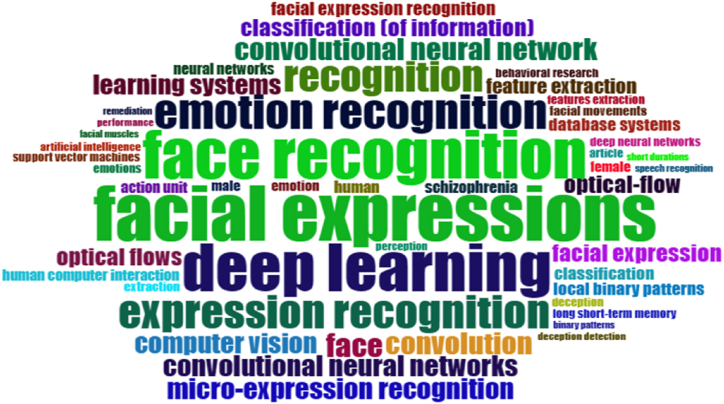

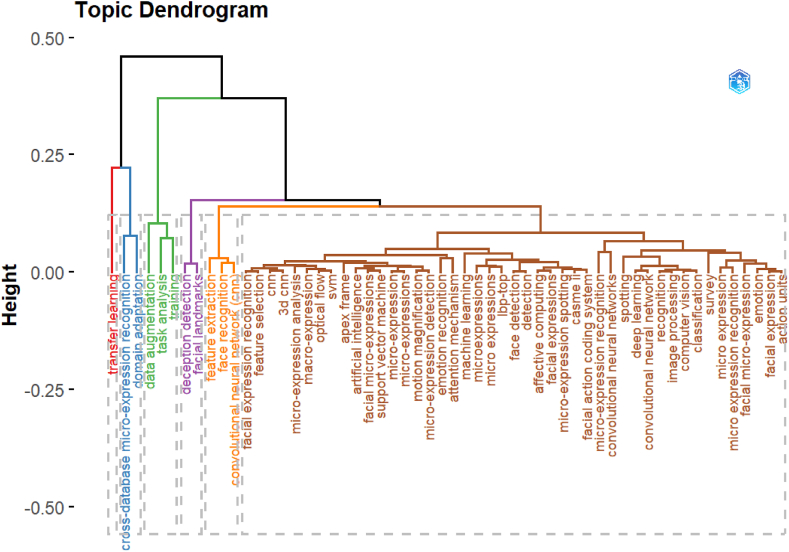
(a). Word cloud for the most relevant keywords in micro expression recognition publications (b). Dendrogram of Authors keywords.

The words most commonly employed by researchers in the keywords section were also assessed, given their tendency to convey the central focus of the research. Fig. 7a depicted that deep learning, facial expression recognition, and emotion recognition were among the most frequently used terms. The substantial and conspicuous font visibility of the keywords is apparent, as demonstrated by their large and bold font. Fig. 7b illustrated the corresponding analysis and clustering dendrogram for the aforementioned keywords. A dendrogram is a diagram that depicted the word's hierarchical relationship. A dendrogram is primarily used to determine the best way to assign words to clusters. Fig. 7b depicted the hierarchical clustering of word observations using a dendrogram. Focusing on the height at which any two words were connected together was essential for dendrogram comprehension. Fig. 7b showed that micro expression and facial micro expression recognition were more similar in the brown cluster, with the shortest link connecting them. The height of the blue and green clusters, on the other hand, was greater, indicating the difference between the words.

#### 3.1.7 Network analysis

This bibliometrics study was conducted using Bibliometrix (R-based software for mapping literature), for quantitative bibliometric and scientometric research, the bibliometrix R-package (http://www.bibliometrix.org) offers a number of tools. It is written in R, an open-source language, environment, and ecosystem. The best reasons to choose R over other languages for scientific computing may be found in its extensive, powerful statistical algorithms, easy access to excellent numerical routines, and integrated data visualization tools. Similarly, VOSViewer [41] is a visualization tool specifically designed for constructing and visualizing bibliometric networks. It is widely used in the academic and research community for analyzing relationships between scholarly entities such as authors, keywords, and publications. The mainstream of the data analysis was conducted with the help of Bibliometrics, based on Bibliographic Cooccurrence Analysis (BCA), Bibliographic Coupling, Co-Authorship, Quotation, and Co-Citation Analysis. To analyze the data and report the results, the software manuals of Guleria and Kaur [48] were used.

In network visualizations of scientific data, various entities such as authors, keywords, nations, and organizations have been depicted by emphasizing prominent nodes [49]. It is a rare occurrence to observe two entities, such as authors and publishing, being portrayed in a single map. The size of the node (circle) in network mapping represents the measured value of these elements. The significance of the node is directly proportional to its numerical value, which primarily indicates the number of citations or occurrences in an article. The linkages (edges) that connect the nodes reflect their relationship. The strength (weight) value of connections determines the association between two nodes, with a higher total link strength (TLS) value indicating a stronger association. Consequently, if a node represents the frequency of appearance of an article, the links between nodes represent the number of references exchanged. Therefore, as the number of nodes increases, the TLS value (weight) also increases. Additionally, the color and position of nodes in a network map serve as relevant indicators. When two articles (nodes) are in close proximity, they appear linked and share a greater number of references. The usage of the same color signifies that the articles belong to the same category.

#### 3.1.8 Co-occurrence analysis (documents)

**Fig. 8:**
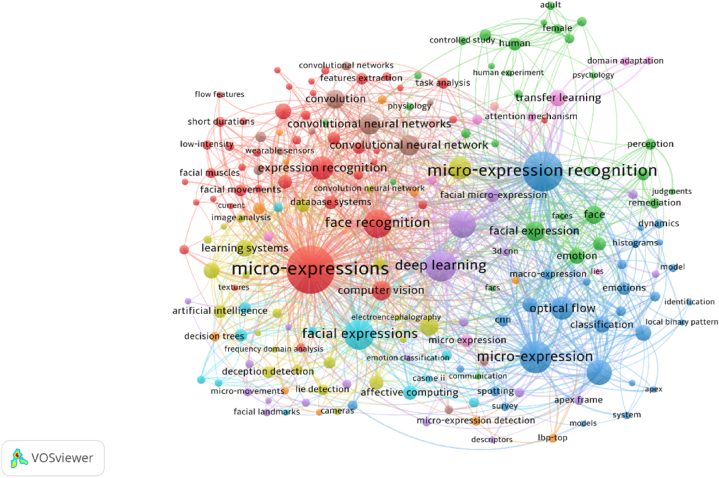

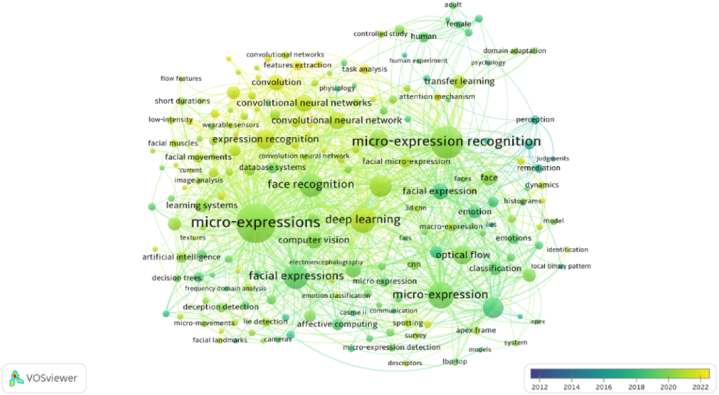
(a). A network representation of all keywords. (b). All keywords are visualized as an overlay.

The keyword sections of the author, for example, provide information in a distinctive manner. The term "co-occurrence" refers to how frequently a term appeared in particular publications [50]. The strength of a word's overall length determines how many times it appeared in a text. The size of the node is directly proportional to the frequency of the words; for instance, a larger node size indicates a higher prevalence of the term. A thicker line linking two or more terms indicates their proximity to a particular cluster. To better grasp the intellectual terms cited by diverse professors, co-occurrence analysis was used. In total, 1410 author keywords and 1989 index keywords were discovered. Table 1 displays the TLS value of the ten top authors and index keywords. "Micro-expression recognition, micro-expression," and "deep learning" were identified as the most essential terms in both groups. Fig. 8a depicted the network visualization of all keywords. Micro-expression recognition, micro-expressions, and deep learning were the most prominent nodes. Keywords were also investigated between the years of 2012 and October 2022. The overlay visualization of all keywords is shown in Fig. 8b. A close examination of the graph revealed that the keywords used most frequently in late 2022 were expression recognition, convolutional neural network, micro-expression analysis, task analysis, and feature extractions.

**Table 1:** The outcomes derived from the co-occurrence analysis conducted on author and index keywords.

Author Keyword	TLS	Index Keyword	TLS
Micro-expression recognition	203	Micro-expression	1128
Deep learning	174	Face recognition	445
Micro-expression	169	Facial expressions	434
Optical flow	125	Deep learning	381
Emotion recognition	84	CNN	282
Features Extraction	81	Computer vision	203
Recognition	65	Classification	178
Affective computing	33	Learning system	181
Transfer learning	49	Image enhancement	55
Task analysis	46	semantics	47

#### 3.1.9 Bibliographic coupling

The early data screening of the top ten publications and conferences is shown in Table 2. The rankings of journals and conferences have been determined by fractional TLS values. Numerous studies on ME recognition across various fields were found, thus researchers were not restricted to a few journals only. Among the journals and conferences used by researchers in this subject were Pattern Recognition Letters, Plos One, ACM International conference proceeding, sensor, IEEE Access, frontiers in psychology, neurocomputing, and lectures notes in computer science. In addition, at least six studies in this topic have been published in various journals and conferences. The top conference in this field is Lecture Notes in Computer Science, which is followed by the IEEE Transaction on Affective Computing, ACM International Conference Processing, and Neurocomputing.

**Table 2:** The following list comprises the ten most esteemed journals and conferences, selected based on their TLS.

Name of the Journals conferences	Documents	Citation	TLS
lecture notes in computer science	24	60	81
IEEE transaction on affective computing	20	822	35
Neurocomputing	17	411	4
Frontiers in phycology	14	153	8
Multimedia Tools and Application	15	171	5
IEEE Access	13	166	4
Pattern Recognition letters	11	52	11
Sensors	10	32	10
ACM International conference proceeding	21	17	34
Plos One	6	449	0

**Fig. 9:**
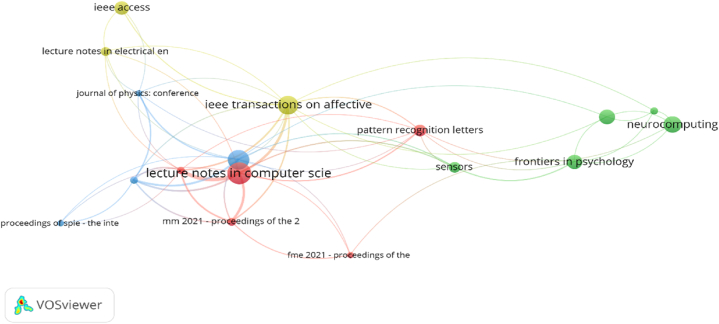

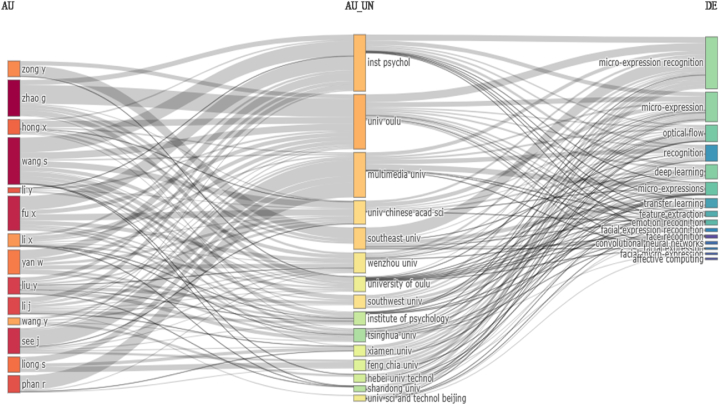
(a). Bibliographic coupling for journals and conferences. (b). Triple field plot of authors, affiliation, and keyword.

The bibliographic coupling for journals and conferences, with a minimum of five articles and five citations per article, is depicted in Fig. 9a.

In bibliographic coupling, TLS values were used to rank authors, organizations, and countries. The strength of interconnectedness between two nodes (authors, organizations, and countries) was determined via TLS. After applying the threshold criterion, Table 3 displayed the ten top authors, organizations, and nations. Wang S obtained the greatest impact value, followed by Zhang L and Buhari A, indicating that their contribution to micro-expression recognition was greater than that of the other authors and that their publications were more valuable. The Chinese Academy of Sciences was regarded as the most prominent organization working in this area among the top ten.

**Table 3:** The top ten contributing authors, countries, and organizations were subjected to a bibliographic coupling study.

Authors (TLS)	Organizations (TLS)	Countries (TLS)
Wang S (5)	Chinese Academy of Sci (123)	China (1836)
Zhang L (4)	University of Oulu (86)	United Kingdom (1044)
Buhari A (3)	Multimedia university (49)	Finland (783)
Li J (3)	Wenzhou University (38)	Malaysia (389)
Verma M (3)	Xiamen University (32)	India (385)
Happy S (2)	Monash university (30)	United States (379)
See J (2)	Tsinghua University (19)	Australia (282)
Lu H (1)	Southeast University (18)	France (282)
Ade gun I (0)	Shandong University (18)	Taiwan (161)
Allaert B (0)	Xian jiaotong University (16)	Indonesia (143)

Multimedia University Malaysia was ranked second, followed by the University of Oulu in the United States. TLS values shown in Table 3 showed that the most proficient country was China, followed by the United Kingdom, Finland, and Malaysia. It was claimed that people republic of China (PRC) has published more research articles as compared to United Kingdom (UK) and Finland, and that the value of the articles published by PRC was greater than UK and Finland. Surprisingly, some developing countries, such as India and Indonesia, contributed significantly in this area. Fig. 9b showed a triple field plot built using a bibliometric, an R-based tool. The relationship between authors (left), affiliation (center), and authors' keywords (right) was depicted in this graphic. This graphic was based on count values such as the number of publications per author and institute, as well as the frequency with which keywords appear in the database. The sequence layout, on the other hand, would be different. According to the graph, three writers affiliated with the University of Chinese Academy of Science published the majority of articles on micro-expression recognition. The majority of publications were concerned with micro-expression and its key applications (recognition and deep learning). Furthermore, the majority of the publications contain the term transfer learning.

#### 3.1.10 Co-authorship of countries

**Fig. 10:**
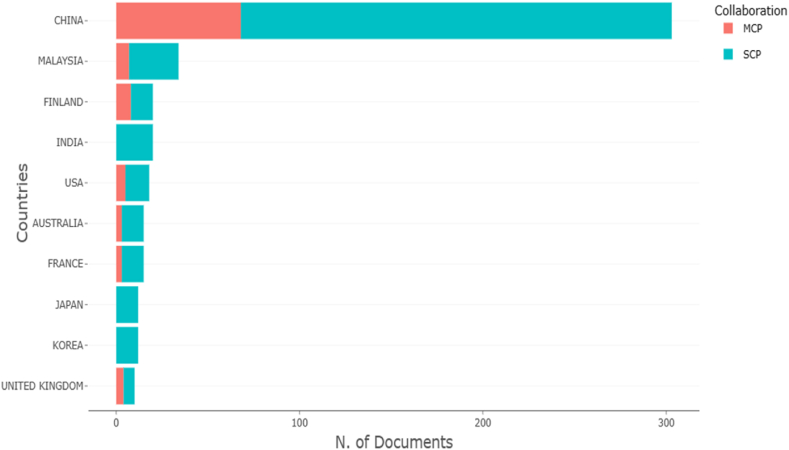
Top ten countries in terms of co-authorship.

Co-authorship analysis investigated how researchers worked within a specific subject. Because co-authorship is a formal form of intellectual collaboration among researchers [51], understanding how academics interact is critical. Academic collaborations are becoming more common as the methodological and theoretical complexity of research increases [52]. Collaborations amongst academics, for example, can improve the clarity and depth of research. Academic collaboration forms a network known as invisible collages, the study of which might aid in the advancement of research projects [53]. These insights can be employed to foster and inspire novel research endeavors undertaken by scholars hailing from underrepresented demographics. For instance, the analysis can provide valuable illumination on the prevalence of concentrated research efforts within the academic community originating from a particular geographical area. Moreover, the analysis facilitates the delineation of collaborations throughout the course of time, enabling researchers to scrutinize the trajectory of intellectual advancement in correlation with collaborative networks. This offers irreplaceable guidance to aspiring researchers regarding the means to establish contact and to collaborate with esteemed and renowned researchers in their respective field. Fig. 10 depicted the top ten countries for co-authorship of country assessments, demonstrating the collaboration of experts from many countries. MCP (multiple country publications) refers to the degree of international collaboration between nations, whereas SCP stands for single country publications, which occurs when all authors are from the same country. According to the co-authorship analysis, China has most affiliations with other nations with 68 MCP and 235 SCP and a frequency of 0.419, followed by Malaysia with 7 MCP and 27 SCP and a frequency of 0.047. Finland's MCP and SCP were 8 and 12, respectively, with frequencies of 0.028.

#### 3.1.11 Co-authorship (authors)

**Fig. 11:**
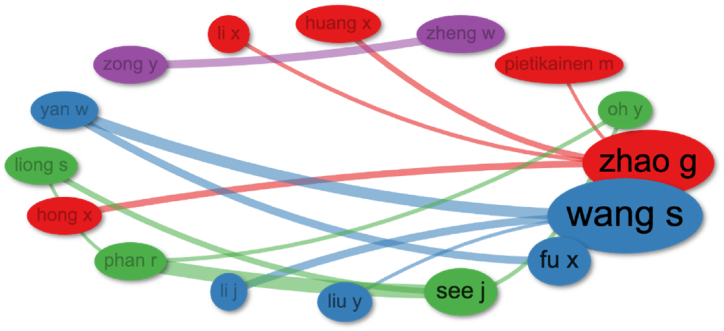

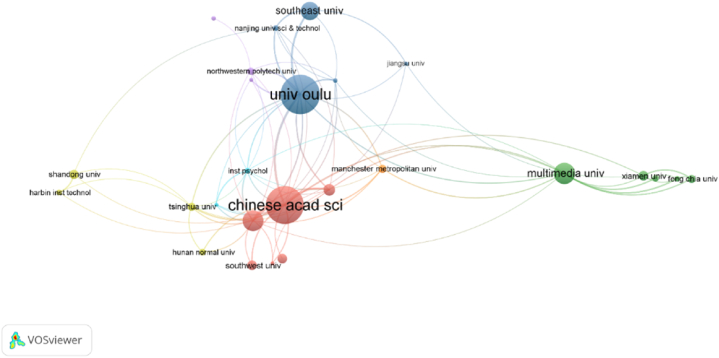

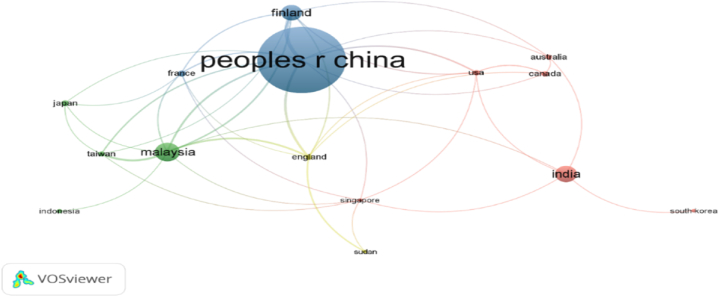
(a). Co-authorship analysis shown the top 4 author clusters. (b). An analysis of co-authorship within organizations. (c). The co-authorship analysis for countries.

A co-authorship analysis was performed to better understand the contributions of the various authors. Co-authorship analysis quantifies the extent to which scientists have published together to enhance knowledge in a scientific topic. Fig. 11a showed the top four authors clustered together with a resolution of 1.0 and a citation threshold of 5. Citation threshold parameter indicated that nodes in the citation network must have a at least 5 citations in common to be considered for clustering together and the resolution parameter influenced the granularity of the clustering. The higher the resolution value results in smaller the more distinct clusters, while the lower value the more inclusive cluster. Fig. 11a clearly demonstrated that the authors in cluster 1 (blue) were Wang S and Fu X, and See J and Phan R were similarly more prolific authors in cluster 2 (green), Zhao G and Hong X were more productive authors in cluster 3 (red), and Wang Z and Zong Y were strong collaborative authors in cluster 4 (purple). The top ten writers in the bibliographic coupling study were also the most notable authors in a few clusters. Furthermore, many authors were from the same region PRC and like to interact with other authors from the same region.

Table 4 presented the co-authorship analysis pertaining to the organizations. In the case of the organization, only two clusters were taken into consideration. Cluster 1 (red) comprises four colleges and exhibits a total of 19 link values. On the other hand, Cluster 2 (green) demonstrated an average link value of 11. Compared to other analysis, co-authorship for an organization has not been harmonized as it contained threshold values for a minimum number of documents and citations of an organization which is challenging to meet simultaneously. Secondly, organizations included long names, including the school, university, city, and country. That is why the organization's name may not have a consistent format. Co-authorship analysis for an organization is shown in Fig. 11b.

**Table 4:** Connections and TLS for co-initiation examination for an association.

Cluster 1 (Red)	Links	TLS
Chinese Academy of Science	19	81
Wenzhou University	9	27
Tsinghua University	9	19
Institute of Physics Chinese academy of science	10	21
Cluster 2 (Green)		
University of Oulu	14	65
Xi'an Jiaotong University	8	14
Nanjing University of Science and Technology	6	14
Southeast University	4	17

As shown in Fig. 11c and Table 5, the co-authorship analysis for 14 nations may be separated into four groups. Cluster 3 (Blue) is the most powerful, including the People's Republic of China, Finland, and France as members. Furthermore, Malaysia and Taiwan are in cluster 2 (green), Australia and Singapore are in cluster 3 (red), while England and Sudan are in cluster 4 (yellow).

**Table 5:** Connections and TLS for co-author examination for every country.

Cluster 1 (Red)	Links	TLS	Cluster 2 (Green)	Links	TLS
Singapore	7	7	Malaysia	9	29
USA	6	11	Taiwan	5	14
Australia	4	4	Japan	4	4
India	5	5	Indonesia	1	1
South Korea	1	1	Cluster 3 (Blue)		
Cluster 4 (Yellow)			People R China	10	43
England	7	21	Finland	7	29
Sudan	2	4	France	6	10

#### 3.1.12 Citation analysis

The analysis of citations serves as a valuable tool for evaluating the research standing of authors, countries, and organizations. In this particular study, citation analysis was employed to rank papers based on both global and local citations. The global citation rating of papers can be found in Table 6. A global citation (GC) pertains to the quantity of citations garnered within the database, and this metric functions as an indicator of an article's prominence within its corresponding discipline. Notably, Yan WJ et al. [9], garnered the highest number of global citations (365), followed by Davison et al. [54], Kemeny et al. [55], and Liu et al. [20], who received 229, 196, and 177 citations respectively. It is worth mentioning that the updated micro-expression-based database developed by Yan WJ et al. (2014) could potentially account for their significant citation count.

**Table 6:** Top Five most frequently cited articles worldwide in micro-expression recognition.

Author	Title	Journal names	GC	GCPY
Yan et al. (2014) [9]	CASME II: An Improved Spontaneous Micro-Expression Database and the Baseline Evaluation	Plos One	365	36.50
Davison et al., 2018 [54]	SAMM: A Spontaneous Micro-Facial Movement Dataset	IEEE Transactions on Affective Computing	229	38.17
Kemeny et al., 2012 [55]	Contemplative/emotion training reduces negative emotional behavior and promotes prosocial responses.	Emotion	196	16.33
Liu et al., 2016 [20]	A Main Directional Mean Optical Flow Feature for Spontaneous Micro-Expression Recognition	IEEE Transactions on Affective Computing	177	22.13
Yan et al., 2013 [3]	How Fast are the Leaked Facial Expressions: The Duration of Micro-Expressions	Journal of Nonverbal Behavior	168	15.27

#### 3.1.13 Co-citation analysis

**Fig. 12:**
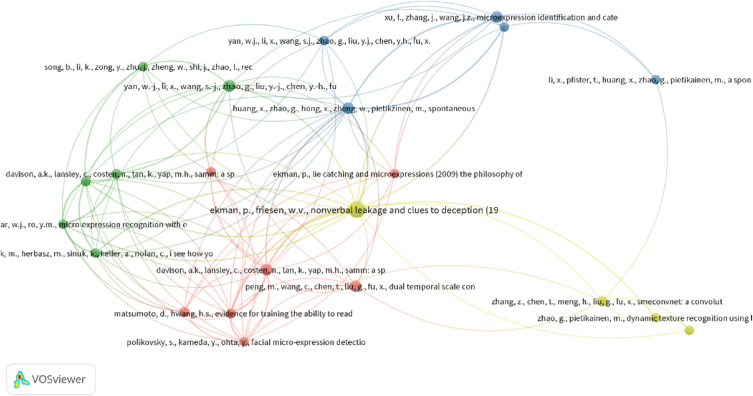
References based Co-citation analysis.

Co-citation occurs when two articles are cited in the reference list of a third publication [56]. Journal publications are represented by nodes in a citation analysis, while co-citations are represented by edges [57]. As a result, as the number of edges increases, so does the co-citation value. The greater the co-citation value, the more closely these papers are related and work in the same subject. We also discovered that papers with at least one reference were present in that analysis. Because it is difficult to evaluate all of the articles, we chose the top 50 papers and established criterion of at least five citations. Fig. 12 depicted the analysis of reference co-citation. Four clusters were present, each having 104 connections and 150 TLS. This showed that the articles in each cluster belong to the same category and have the same reference list. Furthermore, with 20 links, 34 TLS, and 23 citations, Ekman et al., in cluster 4 (red) has the most significant article, followed by Huang et al., in cluster 3 (blue), with 16 links, 23 TLS, and 8 citations. This demonstrated that the overwhelming majority of articles incorporated these binary articles within their reference list.

## 4 Limitation

In the current study, bibliometric research and citation analysis were conducted using the WoS and Scopus databases, two trustworthy sources of worldwide peer-reviewed publications. Despite its benefits, the present study includes certain drawbacks. First, the impact of self-citations was not completely disregarded. Secondly, the bibliometric analysis is done on micro-expression recognition, in which all the proposed algorithms are considered. Thirdly, the literature that is returned depends on the search terms, which cannot be guaranteed to return any alternative terms used by other scholars. ME has been studied in various disciplines, including psychology, computer science, and communication. Restricting the search to a specific discipline may overlook relevant contributions from another field. The topic was limited to computer science and information and communication Engineering (I.C.E). Limiting the search to specific languages may result in the exclusion of relevant studies published in other languages. In our study our focus was on English-language conferences, journals, and review publications. Different authors may use various terms to describe micro-expressions. The choice of keywords may influence the retrieval of relevant articles. Variability in terminology can lead to missed articles or the inclusion of irrelevant ones. The information gathered would undoubtedly grow if non-English documents were included. Because of the constraints, it is possible that the results do not accurately reflect all of the papers on micro-expression recognition that are currently available in databases. Additionally, the use of citation networks as a gauge of effect and quality may be subjected to criticism. Through the comprehensive and systematic inclusion of all document kinds and languages, the comparative utilization of various databases, and the use of bibliometric mapping tools, these constraints could serve as further research fields for bibliometric analysis on similar studies.

## 5 Conclusion

This research endeavor constituted a comprehensive examination of published articles pertaining to the domain of micro expression recognition. It encompassed fundamental data derived from antecedent literature, encompassing the quantity of articles, yearly publications, and topical domains, and scrutinized the most impactful authors, articles, journals, and countries through the utilization of VOSviewer and R-based tools. The proposed study revealed that the majority of articles were about micro expression and facial micro-expression recognition. In terms of the subject domain, the research hotspots were micro-expression recognition and deep learning, followed by a core application of micro-expression recognition (optical flow, emotion recognition, features extractions, affective computing, transfer learning). Furthermore, the author's keywords included the fundamental terms of ME recognition, whereas the index keywords included broad terms. When it comes to journals, IEEE Transactions on Affective Computing has published the most articles as compared to the other journals. In terms of countries, China published the most articles and was also leading in terms of authorship and co-citation analysis using TLS and citation values. It was also noticed that majority of the authors and institutions were from China and Malaysia, with micro expression recognition being the leading research area. In future work, an algorithm which take less training time when train on micro expression datasets can be developed. Secondly, the micro expression dataset is highly imbalanced so over coming this problem an algorithm can be improved to overcome this issue.

## CRediT authorship contribution statement

**Adnan Ahmad:** Writing – original draft, Software, Conceptualization. **Zhao Li:** Investigation, Formal analysis. **Sheeraz Iqbal:** Writing – review & editing, Project administration, Investigation. **Muhammad Aurangzeb:** Formal analysis, Data curation. **Irfan Tariq:** Visualization, Validation. **Ayman Flah:** Investigation, Funding acquisition, Data curation. **Vojtech Blazek:** Visualization, Validation. **Lucas Prokop:** Investigation, Formal analysis.

## Declaration of competing interest

The authors declare the following financial interests/personal relationships which may be considered as potential competing interests:This work Aknowldgment: This paper was supported by the following project TN02000025 National Centre for Energy II. The authors declare there is no conflict of interest.
